# Cytosine methylation is a conserved epigenetic feature found throughout the phylum Platyhelminthes

**DOI:** 10.1186/1471-2164-14-462

**Published:** 2013-07-09

**Authors:** Kathrin K Geyer, Iain W Chalmers, Neil MacKintosh, Julie E Hirst, Rory Geoghegan, Mathieu Badets, Peter M Brophy, Klaus Brehm, Karl F Hoffmann

**Affiliations:** 1 Institute of Biological Environmental and Rural Sciences (IBERS), Aberystwyth University, Penglais Campus, Aberystwyth SY23 3FG, United Kingdom; 2 School of Environmental Science and Development, North-West University, Potchefstroom Campus, Potchefstroom 2520, South Africa; 3 Institute of Hygiene and Microbiology, University of Würzburg, Würzburg, Germany

**Keywords:** Platyhelminthes, DNA methylation, Epigenetics, Flatworm, 5-methyl cytosine

## Abstract

**Background:**

The phylum Platyhelminthes (flatworms) contains an important group of bilaterian organisms responsible for many debilitating and chronic infectious diseases of human and animal populations inhabiting the planet today. In addition to their biomedical and veterinary relevance, some platyhelminths are also frequently used models for understanding tissue regeneration and stem cell biology. Therefore, the molecular (genetic and epigenetic) characteristics that underlie trophic specialism, pathogenicity or developmental maturation are likely to be pivotal in our continued studies of this important metazoan group. Indeed, in contrast to earlier studies that failed to detect evidence of cytosine or adenine methylation in parasitic flatworm taxa, our laboratory has recently defined a critical role for cytosine methylation in *Schistosoma mansoni* oviposition, egg maturation and ovarian development. Thus, in order to identify whether this epigenetic modification features in other platyhelminth species or is a novelty of *S. mansoni*, we conducted a study simultaneously surveying for DNA methylation machinery components and DNA methylation marks throughout the phylum using both parasitic and non-parasitic representatives.

**Results:**

Firstly, using both *S. mansoni* DNA methyltransferase 2 (SmDNMT2) and methyl-CpG binding domain protein (SmMBD) as query sequences, we illustrate that essential DNA methylation machinery components are well conserved throughout the phylum. Secondly, using both molecular (methylation specific amplification polymorphism, MSAP) and immunological (enzyme-linked immunoabsorbent assay, ELISA) methodologies, we demonstrate that representative species (*Echinococcus multilocularis*, *Protopolystoma xenopodis*, *Schistosoma haematobium*, *Schistosoma japonicum*, *Fasciola hepatica* and *Polycelis nigra*) within all four platyhelminth classes (Cestoda, Monogenea, Trematoda and ‘Turbellaria’) contain methylated cytosines within their genome compartments.

**Conclusions:**

Collectively, these findings provide the first direct evidence for a functionally conserved and enzymatically active DNA methylation system throughout the Platyhelminthes. Defining how this epigenetic feature shapes phenotypic diversity and development within the phylum represents an exciting new area of metazoan biology.

## Background

The Platyhelminthes (flatworms) contain a diverse group of acoelomate, triploblastic organisms. While debate continues as to the evolutionary relationship of the 100,000 or so extant species within this phylum [1], the general consensus remains that these metazoans consist of four major clades: the monophyletic classes Cestoda (endoparasitic tapeworms), Monogenea (mostly ectoparasitic forms) and Trematoda (endoparasitic flukes) as well as the paraphyletic ‘Turbellaria’ (mostly free-living species). As tractable examples of development and self-regeneration (e.g. turbellarians and cestodes) or as causative agents of aquaculture, veterinary and biomedically-relevant parasitic diseases (e.g. monogeneans, trematodes and cestodes), the platyhelminths are an intensively studied invertebrate group. These research projects have fuelled numerous ‘omics’ investigations, which in turn, have yielded genetic information pertinent to our current understanding of platyhelminth development, parasite biology, metazoan evolution and adaptation. However, the likely considerable role of ‘epigenetics’-based mechanisms in the regulation of these diverse biological processes has yet to be fully investigated [2].

Epigenetics explains how heritable (and potentially reversible) changes in gene expression, caused by mechanisms that do not alter the underlying genetic code, contribute to phenotypic diversity within individuals of a population. Amongst the four major types of epigenetic processes (DNA methylation, histone modifications, miRNA mediated transcriptional regulation and nuclear gene repositioning) found within eukaryotes, DNA methylation is the most highly studied. DNA methylation affects phenotypic diversity by regulating allelic exclusion [3], gene expression [4], embryogenesis [5] and repetitive element silencing [6]. Metazoan DNA methyltransferases (DNMT1, DNMT2, DNMT3a/3b [7]) catalyse this feature by transferring a methyl group (CH_3_) from S-adenosylmethionine (SAM) to the 5-carbon (C5) position of cytosine in gDNA. These ‘epigenetic marks’ are subsequently recognised by methyl-CpG binding domain proteins (MeCP2 and MBD1-4) and converted into signals necessary for generating phenotypic diversity [8]. Together, DNMTs and MBDs (complexed with other proteins [9]) comprise the core metazoan DNA methylation system found in both vertebrate and invertebrate species.

Historically, DNA methylation was not thought to occur in the phylum Platyhelminthes. This belief was based on a small number of studies, which used rather imprecise methodologies or only sampled restricted developmental lifecycle stages or species [10-12]. However, by utilising a complementary suite of more sensitive technologies and surveying seven distinct lifecycle stages, Geyer *et al.* recently challenged this long-standing dogma in epigenetics and clearly demonstrated a functional role for DNA methylation in the developmental biology of *S. mansoni*[13]. In this current investigation, we extend findings of *S. mansoni* DNA methylation to other representative platyhelminth species. We report, for the first time, that DNA methylation (on cytosine residues) and essential DNA methylation machinery components (DNMTs and MBDs) are indeed present in both parasitic and free-living flatworm species. The evolutionary conservation of this epigenetic mark in the developmental progression of monophyletic- (parasitic Cestoda, Monogenea and Trematoda) and paraphyletic – (free-living ‘Turbellaria’) platyhelminths [1,14] that inhabit different ecological niches and employ diverse trophic strategies awaits further investigations.

## Results/discussion

### The Platyhelminthes contain conserved DNMT2 DNA methyltransferases

As a phylum, the Platyhelminthes are predominantly studied due to their disease causing potential [15] and/or their suitability as models for investigating regenerative/stem-cell biology [16-18]. Recent genome sequencing- and functional genomics- efforts have led to rapid advances in our understanding of the molecular processes underpinning platyhelminth development, anthelmintic resistance, phenotypic plasticity and host interactions. However, the considerable role of epigenetic-based mechanisms contributing to these phenomena has yet to be fully investigated [2]. Indeed, only recently has a function for cytosine methylation been demonstrated in the developmental biology of a platyhelminth species (*S. mansoni*; [13]). Here, a role in schistosome egg production was associated with the transcriptional co-regulation of key schistosome DNA methylation machinery components (SmDNMT2 and SmMBD) that mirrored the detection of genomic 5-methyl cytosine (5mC). Collectively, these data indicated that schistosomes encode a DNA methylation machinery responsible for epigenetic alterations to genome structure and that this process contains components functionally similar to those described in other eukaryotes [7]. Therefore, to investigate whether DNA methylation is uniquely found within the *Schistosoma* or, rather, features as a conserved regulator driving genomic/phenotypic diversity within other parasitic or free-living platyhelminths, we conducted an investigation searching for essential epigenetic mediators (SmDNMT2/SmMBD homologs) and corresponding epigenetic marks (cytosine methylation) throughout the phylum.

**Figure 1:**
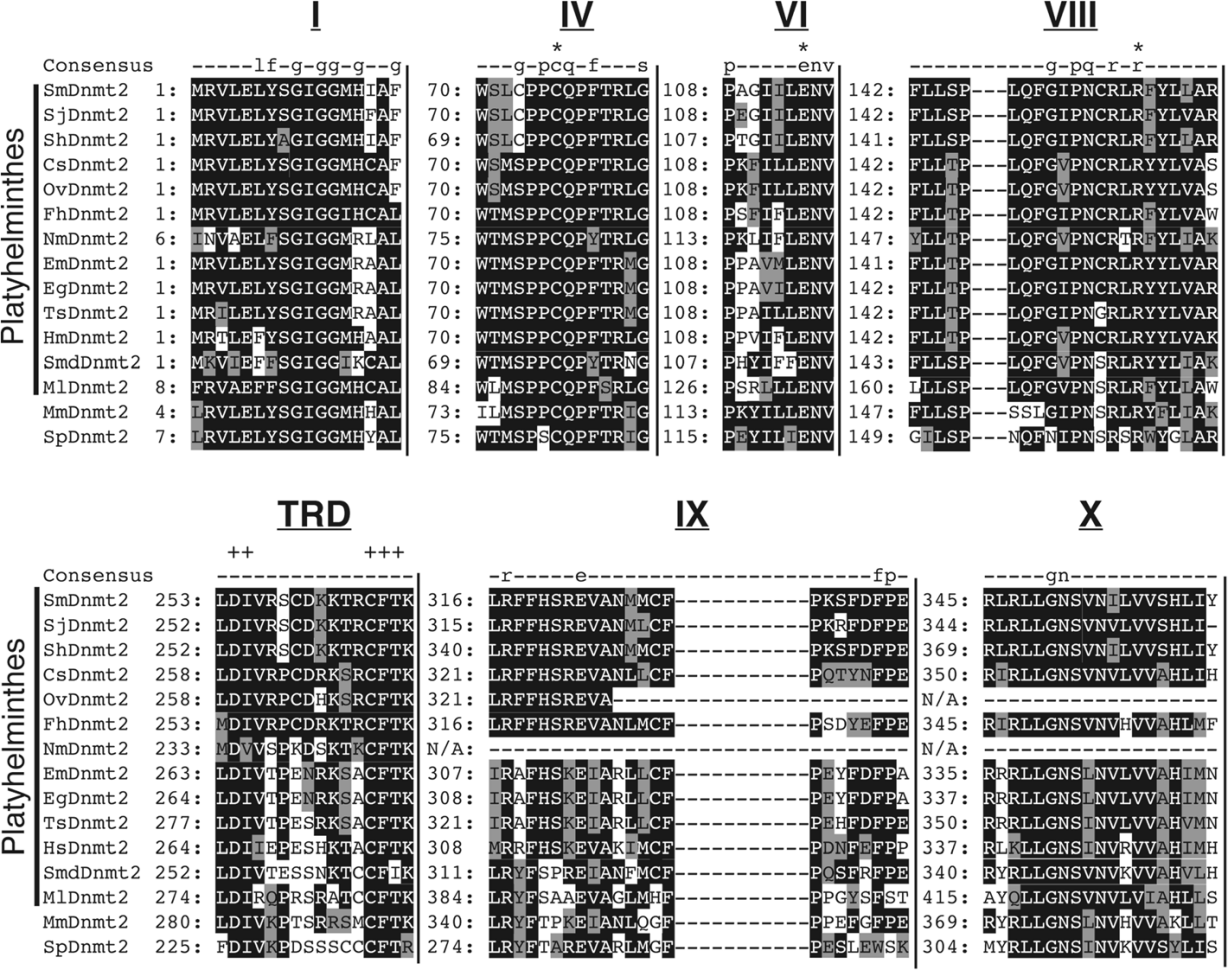
**Platyhelminth DNMT candidates share extensive sequence similarity across the six conserved motifs and the target recognition domain (TRD) of DNA methyltransferase enzymes.** A concatenated multiple sequence alignment of DNMT candidates from *S. mansoni* (Sm), *S. japonicum* (Sj), *S. haematobium* (Sh), *C. sinensis* (Cs), *O. viverrini* (Ov), *F. hepatica* (Fh) *N. melleni* (Nm), *E. multilocularis* (Em), *E. granulosus* (Eg), *T. solium* (Ts), *H. microstoma* (Hm), *S. mediterranea* (Smd) and *M. lignano* (Ml) with *M. musculus* DNMT2 and *S. pombe* PMT1 (NCBI accession numbers are listed in the Methods). The six highly conserved motifs in this catalytic domain are indicated above the alignment in Roman numerals (I, IV, VI, VIII, IX and X) as is the TRD. Numbers at the beginning of each motif represent amino acid positions and at each position the most conserved residues are further shaded in black, semi-conserved residues are highlighted grey and non-conserved amino acids are kept white. The row labelled 'consensus' represents the Pfam consensus sequence of the DNA methyltransferase (PF00145) domain where conserved amino acid residues (50–79%) are indicated by lower-case letters and highly conserved residues (> 80%) are indicated by upper-case letters. Functionally important cysteine (C), glutamic acid (E) and arginine (R) residues within motifs IV, VI and VIII respectively, are indicated by an asterisk (*) above the ‘consensus’ row. DNMT2 specific residues within the target recognition domain (TRD) are indicated by a plus (+) above the ‘consensus’ row. Missing amino acid residues, not present in truncated OvDNMT and NmDNMT candidates are indicated by a ‘N/A’.

SmDNMT2 (NCBI accession number HM991456) and SmMBD (NCBI accession number HM991455) amino acid and nucleotide sequences as well as Pfam searches were used to identify platyhelminth DNMT and MBD candidates in a variety of publically accessible databases [19-24]. Representative species derived from all four platyhelminth classes (Trematoda - *S. japonicum*, *S. haematobium*, *Fasciola hepatica*, *Clonorchis sinensis* and *Opisthorchis viverrini*; Monogenea – *Neobenedenia melleni*; Cestoda – *E. multilocularis, Echinococcus granulosus*, *Taenia solium*, *Hymenolepis microstoma*; Turbellaria – *Schmidtea mediterranea* and *Macrostomum lignano*) contained candidates with high degrees of sequence similarity to SmDNMT2, *Mus musculus* DNMT2 and *Schizosaccharomyces pombe* DNMT2 (PMT1) (Figure 1). Analysis of the thirteen platyhelminth sequences encoding putative DNMT candidates (together with SmDNMT2, MmDNMT2 and PMT1) indicated that they all lacked the large N-terminal regulatory region found in DNMT1 and DNMT3 enzymes [25] as well as an obvious nuclear localization signal (NLS). In contrast, all but two of the putative platyhelminth DNMTs (*N. melleni* and *O. viverrini* being exceptions as they originate from incomplete database entries), contained all ten DNMT-characteristic motifs (I-X) and the target recognition domain (TRD) arranged in the correct order within the C-terminal catalytic, DNA methyltransferase domain (PF00145) [26,27]. Detailed analysis of the six most highly conserved catalytic domain motifs (I, IV, VI, VIII, IX and X) as well as the TRD indicated high sequence conservation across the candidates (Figure 1). Specifically, the enzymatically important cysteine (C76 in SmDNMT2) within the proline/cysteine dipeptide in motif IV is invariant amongst all platyhelminth DNMT candidates. This cysteine mediates covalent bond formation to the target cytosine and is functionally dependent upon the presence of the proceeding proline (P75 in SmDNMT2) [28]. In contrast to PMT1, which is not a functional DNA methyltransferase due to a P to S substitution [29], all platyhelminth DNMT candidates (as well as MmDNMT2) contain this structurally relevant proline residue.

Another invariant region found amongst the platyhelminth DNMT candidates is the glutamic acid/asparagine/valine tripeptide (E114/N115/V116 in SmDNMT2) of motif VI (Figure 1). Within this tripeptide, the glutamic acid residue utilises hydrogen bonding to interact with the target cytosine and is necessary for the stability of the enzyme/target complex [28]. The motif VIII arginine (R157 in SmDNMT2) also facilitates hydrogen bonding to stabilise enzyme/cytosine complexes [28] and is, similar to SmDNMT2 E114, absolutely conserved across the representative platyhelminth DNMTs (Figure 1).

The presence of these six motifs (I, IV, VI, VIII, IX and X) within the C-terminal catalytic domain (PF00145) and the absence of DNMT1 and DNMT3 N-terminal regulatory domains strongly indicate that these new platyhelminth DNMTs are SmDNMT2 homologs. Additionally, conserved aspartic acid/isoleucine dipeptides (D254/I255 in SmDNMT2) and cysteine/phenylalanine/threonine tripeptides (C265/F266/T267 in SmDNMT2) within the TRD (between motifs VIII and IX) add further support to this assertion (Figure 1). These two polypeptide regions are specific to DNMT2 family members and are not found in DNMT1, DNMT3 or bacterial DNA methyltransferases. Importantly, the CFT tripeptide has been additionally proposed to help coordinate target recognition during enzyme interactions [27]. Therefore, strong CFT conservation in all but one (*S. mediterranea* contains a T266 to I266 substitution in the CFT tripeptide) platyhelminth DNMT candidate further supports their inclusion as new DNMT2 family members. While vertebrate DNMT2 family members are now considered predominant tRNA methyltransferases [30], many invertebrate/single-cell eukaryote DNMT2s still retain strong DNA methyltransferase activity [31-34]. The enigmatic role and genomic targets of these new platyhelminth DNMT2s await enzymatic and functional characterisation.

**Figure 2:**
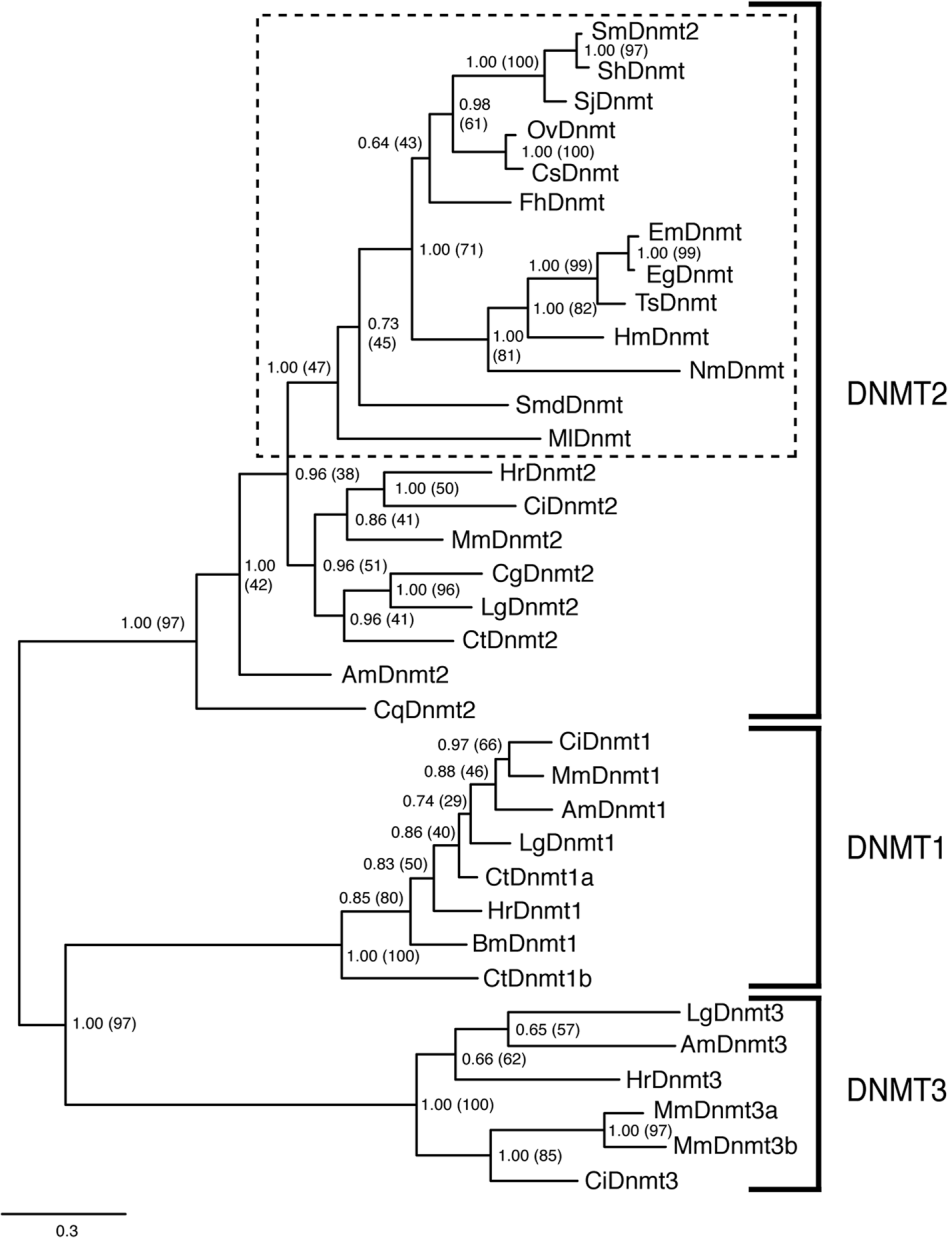
**Platyhelminth DNMTs are novel monophyletic members of the DNMT2 DNA methyltransferase family.** Phylogenetic relationships were inferred from a concatenated multiple sequence alignment (MUSCLE v3.8) of platyhelminth and representative eukaryotic DNMT sequences (NCBI accession numbers are listed in Methods), using Bayesian (implemented in MrBayes v3.1.2) and Maximum Likelihood (implemented in MEGA v5.05) approaches. The figure shows the Bayesian consensus tree illustrated by TreeView, where branch lengths (indicated by scale bar) represent distance between taxa. Only nodes with Bayesian posterior probability support values greater than 0.5 are shown. Node labels within parentheses represent percentage bootstrap support values from Maximum Likelihood analysis (1000 bootstrap replicates performed), while those outside parentheses represent Bayesian posterior probability support values. Platyhelminth sequence names are as listed in Figure 1. Notations Am, Mm, Bm, Ci, Cq, Ct, Cg, Lg and Hr correspond to *A. mellifera*, *M. musculus*, *B. mori*, *C. intestinalis, C. quinquefasciatus*, *C. teleta, C. gigas*, *L. gigantea and H. robusta* sequences respectively. The platyhelminth DNMT2 clade is indicated by a dashed box.

Phylogenetic reconstruction of the platyhelminth DNMT2 family with other characterised DNMTs (e.g. *M. musculus*, *Ciona intestinalis* and *Apis mellifera* DNMT1, DNMT2, DNMT3 members) reveals that, despite minor sequence differences in the platyhelminth homologs, they all cluster in a monophyletic clade (Figure 2, dashed box). These platyhelminth DNMT2 homologs are additionally found within a larger clade that contains MmDNMT2 (mouse), CiDNMT2 (sea squirt), AmDNMT2 (honey bee), CqDnmt2 (mosquito), HrDNMT2 (leech), CtDNMT2 (polychaete annelid), LgDNMT2 (limpet) and CgDNMT2 (oyster) members (Bayesian posterior probability support value = 1; Maximum Likelihood bootstrap value = 97%) adding further evidence for their inclusion as novel DNMT2, but not DNMT1 or DNMT3, DNA methyltransferases. The large genetic distances between DNMT2 family members is likely attributed to their presumed dual substrate (DNA and RNA) specificities [35] when compared to DNMT1 or DNMT3 exemplars (only harbouring DNA methyltransferase activity). Therefore, enzymatic analyses of the platyhelminth DNMT2s remain an important priority to provide a mechanistic explanation for their greater evolutionary divergence when compared to DNMT1 and DNMT3 family members.

### The Platyhelminthes contain conserved MBD2/3 methyl-CpG binding proteins

**Figure 3:**
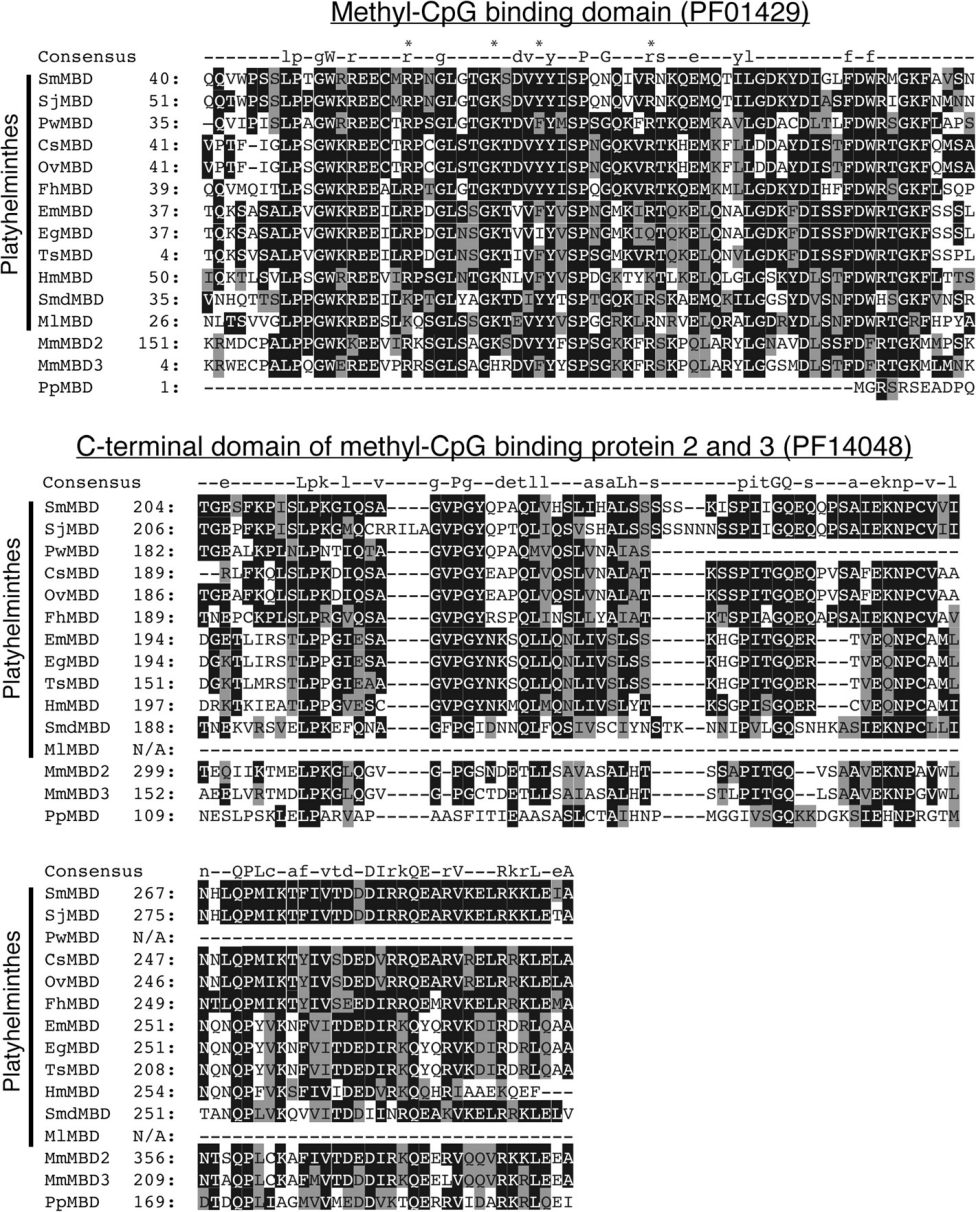
**Platyhelminth MBD candidates share sequence conservation across both Pfam methyl-CpG binding domain (PF01429) and the C-terminal domain of methyl-CpG binding protein 2 and 3 (PF14048) motifs.** Amino acid sequence alignment of the methyl-binding domain (PF01429) and the C-terminal domain of methyl-CpG binding protein 2 and 3 (PF14048) of MBD homologs from *S. mansoni* (Sm), *S. japonicum* (Sj), *P. westermani* (Pw), *C. sinensis* (Cs), *O. viverrini* (Ov), *F. hepatica* (Fh), *E. multilocularis* (Em), *E. granulosus* (Eg), *T. solium* (Ts), *H. microstoma* (Hm), *S. mediterranea* (Smd) and *M. lignano* (Ml) with *M. musculus* MBD2 and MBD3 proteins and the *P. pacificus* MBD protein (NCBI accession numbers are listed in the Methods). Amino acid residues with known functional importance are indicated with an asterisk in upper line. Numbers at the beginning of each line represent amino acid positions and at each position the most conserved residues are further shaded in black, semi-conserved residues are highlighted grey and non-conserved amino acids are kept white. The row labelled 'consensus' represents the Pfam consensus sequence of each domain where conserved amino acids (50–79%) are indicated by lower-case and highly conserved residues (> 80%) by upper-case letters. Missing amino acid residues, not present in the truncated PwMBD and MlMBD candidates, are indicated by a ‘N/A’.

**Figure 4:**
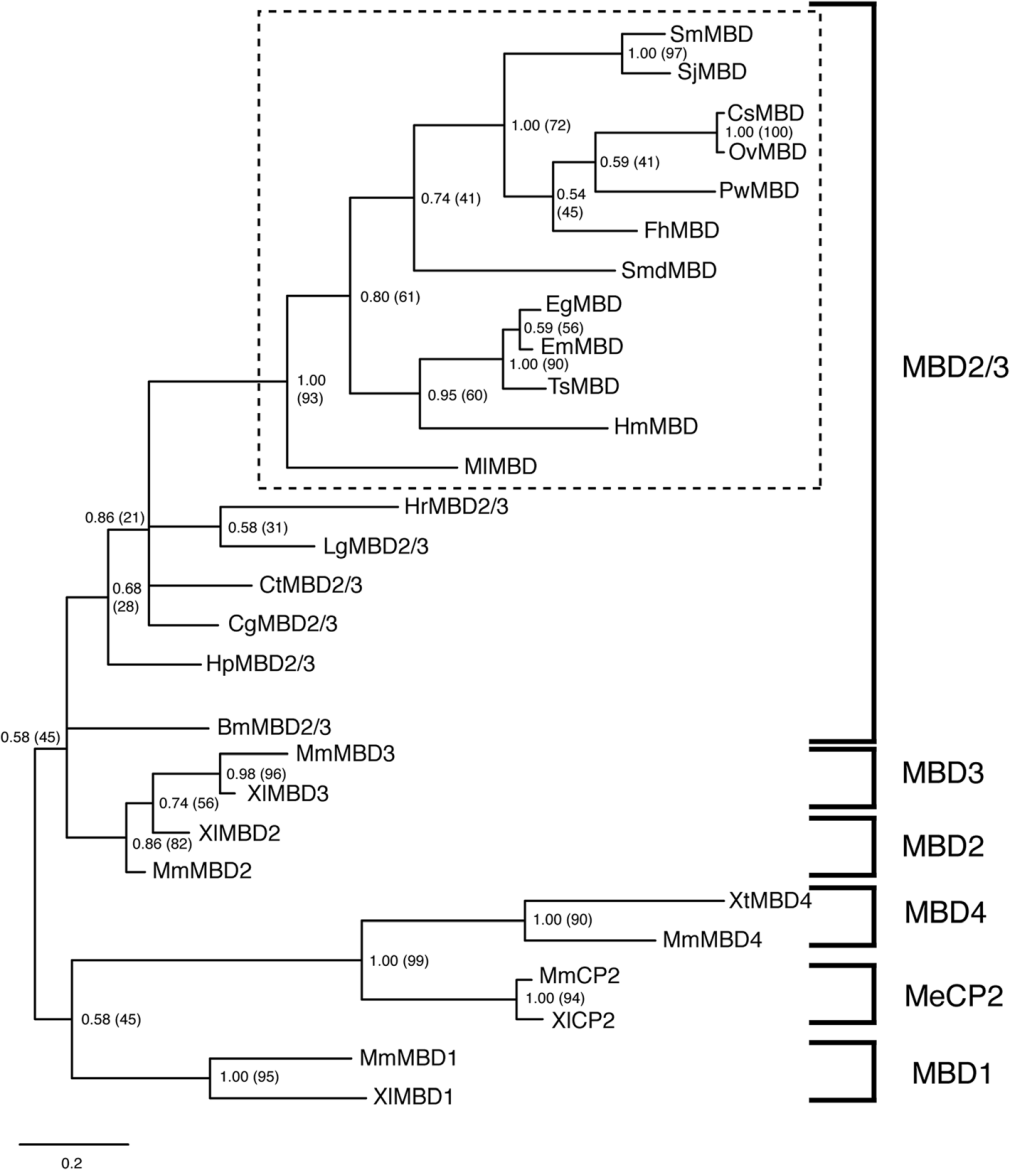
**Platyhelminth MBD candidates are novel members of the invertebrate specific MBD2/3 clade of methyl-CpG binding domain containing proteins.** Phylogenetic relationships were inferred from a multiple sequence alignment (MUSCLE v3.8) of platyhelminth and representative eukaryotic MBD domains (NCBI accession numbers are listed in the Methods) using Bayesian (implemented in MrBayes v3.1.2) and Maximum Likelihood (implemented in MEGA v5.05) approaches. The figure shows the Bayesian consensus tree illustrated by TreeView, where branch lengths (indicated by scale bar) represent distance between taxa; only nodes with Bayesian posterior probability support values greater than 0.5 are shown. Node labels within parentheses represent percentage bootstrap support values from Maximum Likelihood analysis (1000 bootstrap replicates performed), while those outside represent Bayesian posterior probability support values. Notations Hp, Bm, Xl, Xt, Mm, Ct, Cg, Lg and Hr correspond to *H. pulcherrimus*, *B. mori*, *X. laevis*, *X. tropicalis*, *M. musculus*, *C. teleta*, *C. gigas*, *L. gigantea* and *H. robusta* sequences. The platyhelminth MBD2/3 clade is indicated by a dashed box.

Similar to the newly identified platyhelminth DNMT2 homologs, mostly intact MBD candidates were found in the Trematoda (*S. japonicum*, *F. hepatica*, *Paragonimus westermani*, *C. sinensis* and *O. viverrini*), the ‘Turbellaria’ (*S. mediterranea* and *M. lignano*) and the ‘Cestoda’ (*E. multilocularis*, *E. granulosus*, *T. solium* and *H. microstoma*). The exception to our analysis was the ‘Monogenea’, where no MBD candidate was found. It is likely that increased numbers of platyhelminth MBDs (especially within the ‘Monogenea’) will be identified once existing genomes undergo revision [36] or new genomes/transcriptomes are sequenced. Nonetheless, multiple sequence alignment of these platyhelminth MBD candidates with SmMBD, MmMBD2 (mouse) and MmMBD3 (mouse) demonstrated conservation over the 70aa, N-terminal methyl-CpG binding domain (PF01429) and the 97aa, C-terminal domain of methyl-CpG binding protein 2 and 3 (PF14048) (Figure 3). The conservation of these two domains and the fact that only one MBD candidate was found in each of the platyhelminth genomes/transcriptomes suggested that these proteins (in addition to SmMBD) are new members of the ancestral MBD2/3 family [37]. This contention is further supported by the lack of both MBD1 characteristic CxxC Zn-finger motifs [38] and MBD4 characteristic glycosylase DNA repair domains [39]. Furthermore, phylogenetic reconstruction of these novel platyhelminth MBD homologs places them within a large clade (Bayesian posterior probability support value = 0.58; Maximum Likelihood bootstrap value = 45%) containing MBD2/3 (*Hemicentrotus pulcherrimus Bombyx mori*, *Capitella teleta*, *Crassostrea gigas*, *Lottia gigantea* and *Helobdella robusta* MBD2/3s) proteins, and the vertebrate MBD2 and MBD3 homologs (*M. musculus* and *Xenopus laevis*) but outside a distinct clade containing MeCP2 (*X. laevis* and *M. musculus* MeCP2s), MBD1 (*X. laevis* and *M. musculus* MBD1s) and MBD4 (*X. tropicalis* and *M. musculus* MBD4s) exemplars (Figure 4). Collectively, these results strongly support the platyhelminth MBDs as being novel members of the invertebrate specific MBD2/3 family.

Despite sharing greater than 70% amino acid identity, indicative of a gene duplication event [37], mammalian MBD2 is capable of binding methylated cytosine within gDNA whereas MBD3 is not [40]. Therefore, identifying whether these new platyhelminth MBD2/3 proteins share greater sequence similarity to methyl-CpG binding MBD2 or non methyl-CpG binding MBD3 members was investigated. Specific attention was focused on amino acids known to be important for MBD function (i.e. binding to methyl-CpG) and included the following residues: arginine 58 (R58), lysine 66 (K66), tyrosine 79 (Y70) and arginine 80 (R80) (SmMBD2/3 numbering used unless otherwise stated; Figure 3, asterisks above PF01429 consensus sequence). Both K and Y residues are important for MBD function (e.g. Y residue coordinating H-bonding to 5mC [41]) as mammalian MBD3 contains K30 to H30 and Y34 to F34 substitutions (Figure 3) and is unable to bind methylated cytosine [40]. In addition, both R residues also are critical in MBD/target (5mC) interactions as demonstrated by recent X-ray crystallography studies [42,43]. Whereas all platyhelminth MBD2/3s contain conserved K66 and R80 residues, there is variability in the conservation of SmMBD2/3 Y70 and R58 residues. For example, the Y70 amino acid position is maintained in SmMBD2/3, SjMBD2/3, CsMBD2/3 and OvMBD2/3, but is substituted for an F in *P. westermani* MBD2/3 and all of the cestode sequences (*E. multilocularis*, *T. solium*, *H. microstoma* and *E. granulosus*). Furthermore, in *S. mediterranea* SmdMBD2/3, *M. lignano* MlMBD2/3 and *H. microstoma* HmMBD2/3 sequences, a R to K substitution at position 58 is also observed (Figure 3). Based on structural and comparative studies, both of these substitutions likely affect the methyl-CpG binding ability of *P. westermani*, *S. mediterranea M. lignano* and cestode MBD2/3 proteins [41] and may indicate additional/altered functions of these platyhelminth members (perhaps similar to MBD3L1/MBD3L2 [44], MBD3 [45] or other MBD2/3 [46,47] homologs). For SmMBD2/3, SjMBD2/3, CsMBD2/3 and OvMBD2/3 members, containing the full repertoire of functionally-conserved amino acid residues in their methyl-CpG binding domains, it is likely that these proteins bind 5mC. However, as shown for *Drosophila melanogaster* MBD2/3 [48], 5mC recognition of these platyhelminth members may be predominantly in the CpT/CpA context. Evidence to support this contention was recently provided in *S. mansoni*, where cytosine methylation was predominantly found in the CpA context [12,13]. Clearly, further investigations are necessary to extend this locus-specific observation and identify the repertoire of activities mediated by platyhelminth MBD2/3 proteins.

### DNA methylation is found in all four platyhelminth classes

**Figure 5:**
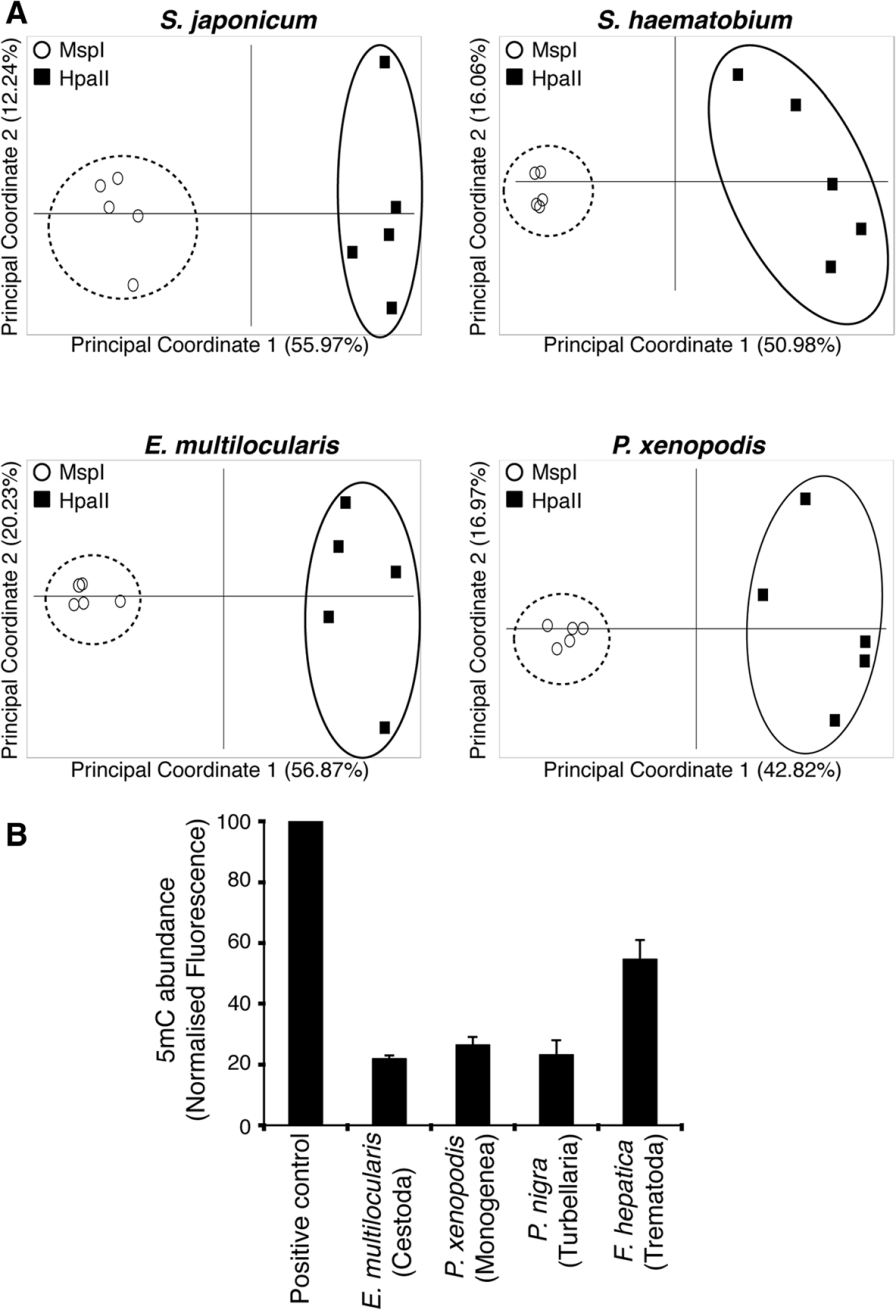
**Global DNA methylation is found in representative species across all four Platyhelminthes classes. A)** MSAP analysis of 5mC in gDNA obtained from *S. japonicum* mixed-sex adults, *S. haematobium* mixed-sex adults, *E. multilocularis* protoscoleces and *P. xenopodis* adults. *Hpa*II (filled squares) and *Msp*I (open circles) isoschizomers were used to restrict gDNA samples. Five replicate samples were analysed for all platyhelminth gDNA samples (biological replicates for mixed-sex adult schistosome populations, biological replicates for adult *P. xenopodis* individuals and technical replicates for *E. multilocularis* protoscoleces). MSAP data (% = the two components explaining the most variability within the dataset) is illustrated by principal coordinate analysis (*Msp*I replicates enclosed by dashed circle; *Hpa*II replicates enclosed by solid oval). **B)** ELISA detection of m5C in *E. multilocularis* protoscoleces, *P. xenopodis* adults, *P. nigra* adults and *F. hepatica* adults. Histograms (+SD, standard deviation) represent mean m5C abundance (n = 3 for each platyhelminth gDNA sample), which was normalised (to both positive and negative control DNA samples) according to the Methods. Data is representative of two independent experiments.

Having established that representative species across all four classes within the Platyhelminthes contain a SmDNMT2 homolog (Figures 1 and 2), we next investigated whether DNA methylation was also conserved across the phylum (Figure 5). Previously, using a variety of technologies including methylation sensitive amplification polymorphism (MSAP), cytosine methylation was found in the *S. mansoni* genome and this epigenetic feature was directly dependent upon the presence of enzymatically active SmDNMT2 [13]. Therefore, the genomes of related schistosome species, *S. japonicum* (containing SjDNMT2) and *S. haematobium* (containing ShDNMT2), were first examined for cytosine methylation (in the CpG context) using the MSAP technique (Figure 5A). Here, principal coordinate analyses (PcoA) of both *S. japonicum* and *S. haematobium* mixed-sex adult parasite gDNA samples, differentially digested with *Msp*I and *Hpa*II isoschizomers, clearly indicated the presence of genomic loci containing 5mC. This finding confirmed that all three medically important schistosome species contained a methylated genome and that MSAP was sensitive enough to detect this epigenetic modification in mixed-sex adult gDNA samples. Similar to *S. mansoni*, it is likely that 5mC detected in mixed-sex adult *S. japonicum* and *S. haematobium* samples is preferentially associated with females where this DNA modification system has previously been shown to regulate oviposition [13]. If this is the case, then selective targeting of *Schistosoma* DNMT2s may represent a pan-genus control strategy useful for novel schistosomiasis interventions.

We next used MSAP to identify cytosine methylation in *E. multilocularis* (containing EmDNMT2, Figures 1 and 2) protoscoleces and *P. xenopodis* (monogenean parasite related to NmDNMT2-containing *N. mellini*, Figures 1 and 2) adults obtained from the urinary bladder of infected *X. laevis* (Figure 5A). Discrete segregation (between *Msp*I and *Hpa*II digested populations) along the first principal coordinate indicated clusters of genomic loci containing this epigenetic modification in both parasitic platyhelminths. Importantly, this is the first molecular evidence for global cytosine methylation in the genomes of both Cestoda and Monogenea species. In contrast to *E. multilocularis* and *P. xenopodis* genomes, we were unable to confidently extend MSAP to detect cytosine methylation in *F. hepatica* (hermaphroditic trematode related to dioecious schistosomes) and *P. nigra* (turbellarian related to SmdDNMT2-containing *S. mediterranea* or MlDNMT2-containing *M. lignano*, Figures 1 and 2) genomes (collected from individual worms). Despite the suggestion of MSAP-detectable DNA methylation in these two species (*Hpa*II and *Msp*I populations are generally separated, Additional file 1), the presence of genetic diversity amongst sampled replicates averts the inference of epigenetic distance. The genetic diversity manifested itself in parasite *Msp*I (methylation insensitive enzyme; indicative of genomic homozygosity/heterozygosity) populations contributing to more variability than parasite *Hpa*II (methylation sensitive enzyme; indicative of methylated genomic loci) populations during multivariate analysis (Additional file 1). The environmental sources of both *F. hepatica* (sheep from different provenances) and *P. nigra* (rocky shores of Llyn-yr-Oerfa) led to sampling of outbred individuals and likely accounted for this genetic diversity.

Therefore, to survey for 5mC in these particular platyhelminth species, an alternative method for detecting 5mC was employed (Figure 5B). This involved an ELISA-based approach, which also was used to successfully identify 5mC in *S. mansoni*[13] and *M. musculus*[49]. Using this methodology, 5mC was found in both *E. multilocularis* and *P. xenopodis* genomes (Figure 5B), confirming the MSAP results for these parasites (Figure 5A) and providing confidence that ELISA-based detection of this genome modification could be translated from *Schistosoma* to other platyhelminth genomes. When subsequently applied to *P. nigra* and *F. hepatica* genomes, clear evidence for 5mC was obtained (Figure 5B). While not exhaustive, this ELISA-based study suggested that digenean trematodes (*F. hepatica*) contain higher levels of 5mC when compared to representative cestode- (*E. multilocularis*), monogenean- (*P. xenopodis*) and turbellarian- (*P. nigra*) species. Quantitative detection of 5mC in additional platyhelminth species (across the four classes within the phylum) would help to clarify this observation.

## Conclusions

The combined presence of conserved DNMT2 and MBD2/3 candidates, as well as cytosine methylation marks throughout the Platyhelminthes, suggests that epigenetic-mediated DNA methylation features in the complex biology of this important phylum. However, it remains to be determined if all species within this phylum have maintained this epigenetic trait or, similar to the Arthropoda [34,50-54] and Nematoda [55], some platyhelminths (especially within the paraphyletic ‘Turbellaria’ [14]) have lost this inheritable mechanism for generating phenotypic diversity. Furthermore, identifying the full enzymatic and target specificities of platyhelminth DNMT2 homologs requires additional investigation. It is currently unknown whether platyhelminth DNMT2s, in addition to DNA, have an affinity for tRNA substrates [30]. Also, the only study to investigate DNA methylation at single base pair resolution within the Platyhelminthes has produced ambiguous results likely due to sampling of *S. mansoni* adult male gDNA only [12]. In previous studies this schistosome developmental form has been shown to contain the lowest level of cytosine methylation throughout the parasite’s lifecycle [13]. Future work will help characterise additional roles of platyhelminth DNMT2s and lead to the identification of genomic positions (e.g. repetitive elements, coding/non-coding genomic loci) targeted by this core DNA methylation machinery component. By doing so in a comprehensive manner (elucidation of DNA methylomes from various lifecycle stages and different platyhelminth species), insights into regenerative-, stem cell- and developmental-biology will be obtained leading to both a greater understanding of the complex life histories within the Platyhelminthes and novel opportunities for control.

## Methods

### Ethical approval

Animal handling, care and subsequent experimentation were performed in compliance with German (Deutsches Tierschutzgesetz,TierSchG, version from Dec-9-2010) and European (European directive 2010/63/EU) regulations on the protection of animals. Ethical approval of the study was obtained from the local ethics committee of the government of Lower Franconia (Regierung von Unterfranken 55.2-2531.01-31/10).

### Platyhelminth material and gDNA isolation

Mixed sex, adult *S. japonicum* and *S. haematobium* worms (blood flukes, class Trematoda) were obtained from perfusion of infected mice and hamsters, respectively [56]. Adult *F. hepatica* worms (liver flukes, class Trematoda) were carefully removed from livers and bile ducts of naturally infected sheep (collected from Randell Parker Foods, Llanidloes, Wales). These parasites were washed six times in pre-warmed (37°C) phosphate buffered saline (PBS, pH 7.3) and cultured (one adult/well in a six well tissue culture plate) in DMEM (Sigma-Aldrich, UK) supplemented with 10% foetal calf serum, 2 mM *L*-glutamine, 100 U/ml penicillin and 100μg/ml streptomycin in an atmosphere of 5% CO_2_ for 48 hr with a 70% media exchange performed after 24 hr. *P. xenopodis* (class Monogenea) adult worms were collected from urinary bladders of infected *X. laevis* originating in Potchefstroom, South Africa. *P. nigra* (class Turbellaria) were collected from stones at the edges of Llyn-yr-Oerfa (Mid Wales) and maintained in a cold-water aquarium for 6 days without food. JAVA, a natural *E. multilocularis* isolate from the liver of an infected cynomolgus monkey (*Macaca fascicularis*; [57]) was passaged intraperitoneally through laboratory jirds (*Meriones unguiculatus*; [58]). *E. multilocularis* genomic DNA (gDNA) was isolated from a pure protoscolex preparation [59] after treatment with pepsin at pH 2 [60] according to a previously established protocol [61]. The remaining platyhelminth gDNA samples were extracted using the Qiagen DNeasy Kit. All gDNA samples were subsequently quantified by a Nanodrop-1000 spectrophotometer. Where possible (*S. japonicum*, *F. hepatica, P. xenopodis* and *E. multilocularis*), gDNA was subjected to host-contamination checks by PCR as previously described [13,62] (Additional file 2).

### Platyhelminth DNMT2 and MBD sequence identification

DNMT2 and MBD members from platyhelminth species were identified by tBLASTn searches of transcripts, predicted genes and genome assemblies of NCBI [19], Welcome Trust Sanger Institute [63] and Gasser Laboratory [20] EST databases as well as *T. solium*[22], *S. mediterranea* (SmedGD v1.3.14; [21]), *M. lignano*[64] and *F. hepatica*[65] gene models/genomic databases using SmDnmt2 IsoformI (NCBI accession no. HM991456) and SmMBD (NCBI accession no. HM991455) as query sequences. A Pfam domain search on SchistoDB v3.0 (beta version; [23]) was performed to identify a DNMT homolog within the genome of *S. haematobium*. Searches were performed on 25-01-12 for *S. japonicum*, *S. haematobium*, *P. westermani, C. sinensis, O. viverrini, N. melleni and S. mediterranea,* 14-11-12 for *F. hepatica* and *M. lignano* and 01-05-13 for *E. granulosus, E. multilocularis, T. solium* and *H. microstoma.* Where multiple ESTs (or ESTs and gene predictions) existed of the same homolog, a consensus sequence was formed. In the cases of *S. haematobium*, *M. lignano*, *F. hepatica*, *E. multilocularis, E. granulosus, H. microstoma* and *T. solium* DNMT homologs, the exon-intron structure of SmDNMT2 IsoformI was used as a reference to identify/correct the platyhelminth DNMT prediction. All exon/intron junctions in these altered gene predictions conformed to the consensus (GT/AG) splice donor/acceptor sequences for eukaryotes. Final amino acid sequences used in the study are listed in Additional file 3.

### Sequence alignments and phylogenetic trees

Multiple sequence alignments of platyhelminth DNMT and MBD candidates were generated using MUSCLE v3.8 [66]. Domain predictions were performed using the Pfam web server [67] to define the limits of the C-5 cytosine-specific DNA methylase domain (DNMT; PF00145), Methyl-CpG binding domain (MBD; PF01429) and C-terminal domain of methyl-CpG binding protein 2 and 3 (MBD_C; PF14048) regions for each sequence. Motif positions represented in the DNMT multiple sequence alignment were derived from a previous report [68]. In addition to the identified platyhelminth candidates, the following sequences were included in the multiple sequence alignments as representative DNMT and MBD members: *M. musculus* MBD2 (NM_010773), *M. musculus* MBD3 (NM_013595), *Pristionchus pacificus* MBD2/3 (AAV85979.1), *M. musculus* DNMT2 (AAC53529.1) and *S. pombe* DNMT2 (CAA57824.1).

For phylogenetic analysis (using Maximum Likelihood and Bayesian approaches) of platyhelminth and non-platyhelminth DNMT members, amino acid sequences were aligned using MUSCLE software with minor improvements in the alignment performed following visual inspection. Non-conserved regions were then removed from the alignment to allow for phylogenetic comparison (during this step the target recognition domain (TRD) and variable regions were removed). In total, 230 amino acid positions were used in the analyses. The non-platyhelminth sequences (and their NCBI accession numbers) included in the DNMT phylogenetic analyses were as follows: *M. musculus* DNMT1 (P13864.5), *M. musculus* DNMT2 *(*AAC53529.1), *M. musculus* DNMT3a (O88508.2), *M. musculus* DNMT3b (O88509.2), *A. mellifera* DNMT1 (NP_001164522.1), *A. mellifera* DNMT2 (XP_393991.3), *A. mellifera* DNMT3 (NP_001177350.1), *C. intestinalis* DNMT2 (XP_002128135.1), *C. intestinalis* DNMT1 (XP_002122948.1), *C. intestinalis* DNMT3a (XP_002123461.1), *Culex quinquefasciatus* DNMT2 (XP_001867327.1), *B. mori* DNMT1 (NP_001036980.1), *C. teleta* Dnmt1a (ELT93682.1), *C. teleta* Dnmt1b (ELU12454.1), *C. teleta* Dnmt2 (ELU13416.1), *C. gigas* Dnmt2 (EKC25033.1), *L. gigantea* Dnmt1 (transcript name: 114987 [69]) *L. gigantea* Dnmt2 (transcript name: 119453 [69]), *L. gigantea* Dnmt3 (transcript name: 171288 [69]), *H. robusta* (transcript name: 116156 [69]), *H. robusta* Dnmt2 (transcript name: 89038 [69]) and *H. robusta* Dnmt3 (transcript name: 162653 [69]). The Bayesian phylogenetic analyses were performed as described previously [70] using MrBayes v3.1.2 [71], the WAG substitution model [72] and a minimum of 1 million generations*.* The log-likelihood score of each saved tree was plotted against the number of generations to determine the point at which the log-likelihood scores of the analyses stabilised*.* Maximum Likelihood analysis was conducted using MEGA 5.0 [73] with the WAG substitution model and 1000 bootstrap replicates. The graphical output of the final Bayesian consensus phylogram was obtained using Figtree v1.3.1 [74]. This tree was then exported into Adobe Illustrator where Maximum Likelihood bootstrap support values for each node were superimposed.

Phylogenetic analyses of the MBD proteins were performed using a MUSCLE alignment restricted to the MBD domain regions of the sequences (as defined by Pfam; PF01429). The non-platyhelminth sequences (and their NCBI accession numbers) included in the MBD phylogenetic analyses were as follows: *M. musculus* MBD1 (NM_013594), *M. musculus* MBD2 (NM_010773), *M. musculus* MBD3 (NM_013595), *M. musculus* MBD4 (NM_010774), *M. musculus* MeCP2 (NM_010788), *X. laevis* MBD1 (NP_001104183.1), *X. laevis* MBD2 (NP_001083787.1), *X. laevis* MBD3 (BAC22082.1), *X. tropicalis* MBD4 (NP_001037916.1), *X. laevis* MeCP2 *(*AAD03736.1), *H. pulcherrimus* MBD2/3 (EU590662), *B. mori* MBD2/3 (BGIBMGA001425-PA; SilkDB [75]), *C. teleta* MBD2/3 (ELT95247.1), *C. gigas* MBD2/3 (EKC32831.1), *L. gigantea* MBD2/3 (transcript name: 112523 [69]) and *H. robusta* MBD2/3 (transcript name: 185546 [69]). Phylogenetic relationships between the 20 members were then inferred in the same way as for the DNMT homologs, using Maximum Likelihood and Bayesian approaches. Maximum Likelihood bootstrap support values for each node (in parentheses) were superimposed onto the Bayesian consensus phylogram using Adobe Illustrator.

### Methylation specific amplification polymorphism (MSAP) analysis of platyhelminth CpG methylation

The MSAP method [76], modified for the detection of CpG methylation in *S. mansoni*[13], was applied to platyhelminth gDNA samples free from host contamination (Additional file 2). Five biological replicates were analysed for *P. xenopodis* (single adults), *F. hepatica* (single adults), *P. nigra* (single adults), *S. haematobium* (mixed-sex adults) and *S. japonicum* (mixed-sex adults) samples. Five technical replicates were examined for *E. multilocularis* protoscolex samples. Following amplicon size determination on an ABI 3100 DNA capillary sequencer (IBERS, Aberystwyth), the raw data was viewed and analysed in GeneMapper® v4.0. GenAlex v6.4 was subsequently employed for multivariate Principal Coordinate Analysis (PcoA).

### ELISA detection of 5mC

The amount of 5mC in 100 ng of selected platyhelminth gDNA samples was analysed using the SuperSense Methylated DNA Quantification Kit (Epigentek) as previously described [13]. Reactions were carried out in triplicates for each platyhelminth sample (*P. nigra*, *F. hepatica*, *P. xenopodis* and *E. multilocularis*) and in duplicates for the negative and positive control (both supplied with the kit). Fluorescent readings (530_ex_/590_em_nm) were obtained using a POLARstar Omega (BMG Labtech, UK) microtiter plate reader and samples were subsequently normalised to controls. The mean of the fluorescent readings obtained from the negative control sample was subtracted from the platyhelminth and positive control values. Subsequently, the fluorescent mean of the positive control values was set at 100% (5mC=100%), and the 5mC abundance in each platyhelminth sample was normalised to this value.

## Competing interests

The authors declare that they have no competiting interests.

## Authors’ contributions

KKG and KFH conceived the project. NM, JIH, RG, MB, PMB and KB collected or contributed materials for the DNA methylation studies. KKG and IWC carried out the multiple sequence alignments and phylogenetic analyses of DNMT and MBD sequences. KKG also performed the MSAP and ELISA analyses of platyhelminth gDNA materials. KKG, IWC and KFH prepared and drafted the manuscript. All authors have read and approved the final manuscript.

## Supplementary Material

Additional file 1 **MSAP analysis of *P. nigra *and *F. hepatica.*** Click here for file

Additional file 2 **Host contamination screen for *S. japonicum*, *F. hepatica*, *E. multilocularis *and *P. Xenopodis.*** Click here for file

Additional file 3 Fasta sequences of retrieved platyhelminth Dnmt and MBD homologs. Click here for file
