# Supplementary material for: Cytosine methylation is a conserved epigenetic feature found throughout the phylum Platyhelminthes
Source: BMC Genomics. 2013 Jul 9;14:462. doi: 10.1186/1471-2164-14-462 (PMC3710501; doi:10.1186/1471-2164-14-462)
Supplement: Additional file 1 — MSAP analysis of P. nigra and F. hepatica. [file 1471-2164-14-462-S1.pdf]

Additional File 1
MSAP analysis of *P. nigra* and *F. hepatica*

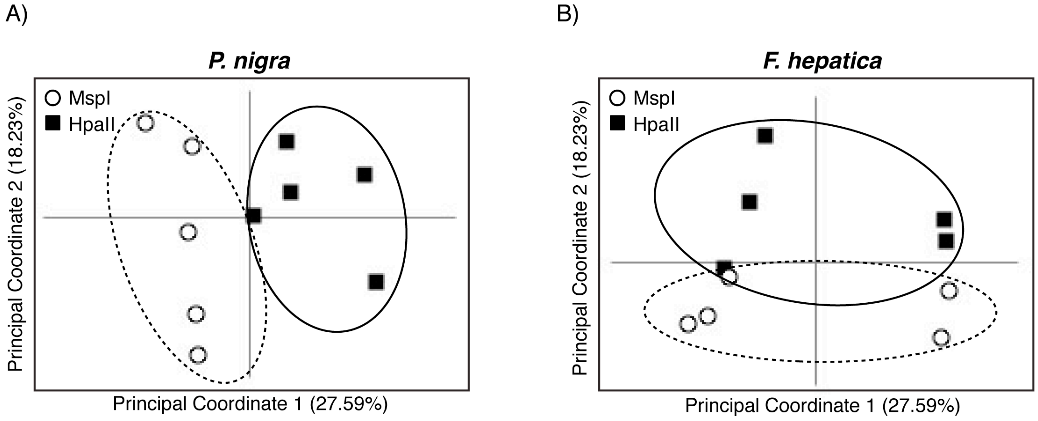

MSAP analysis of m5C in gDNA obtained from *P. nigra* (A) and *F. hepatica* (B) adults. *Hpa*II (filled squares) and *Msp*I (open circles) isoschizomers were used to restrict gDNA samples. Five biological replicate samples each derived from individual worms were analysed for each species. MSAP data (% = the two components explaining the most variability within the dataset) is illustrated by principal coordinate analysis (*Msp*I replicates enclosed by dashed circle; *Hpa*II replicates enclosed by solid oval). The genetic diversity between individual worms of both species is indicated by the variation of the *Msp*I replicates (open circles).
