# Supplementary material for: Cytosine methylation is a conserved epigenetic feature found throughout the phylum Platyhelminthes
Source: BMC Genomics. 2013 Jul 9;14:462. doi: 10.1186/1471-2164-14-462 (PMC3710501; doi:10.1186/1471-2164-14-462)
Supplement: Additional file 2 — Host contamination screen for S. japonicum, F. hepatica, E. multilocularis and P. Xenopodis. [file 1471-2164-14-462-S2.pdf]

Additional File 2

Host contamination screen for *S. japonicum*, *F. hepatica*, *E. multilocularis* and *P. xenopodis*

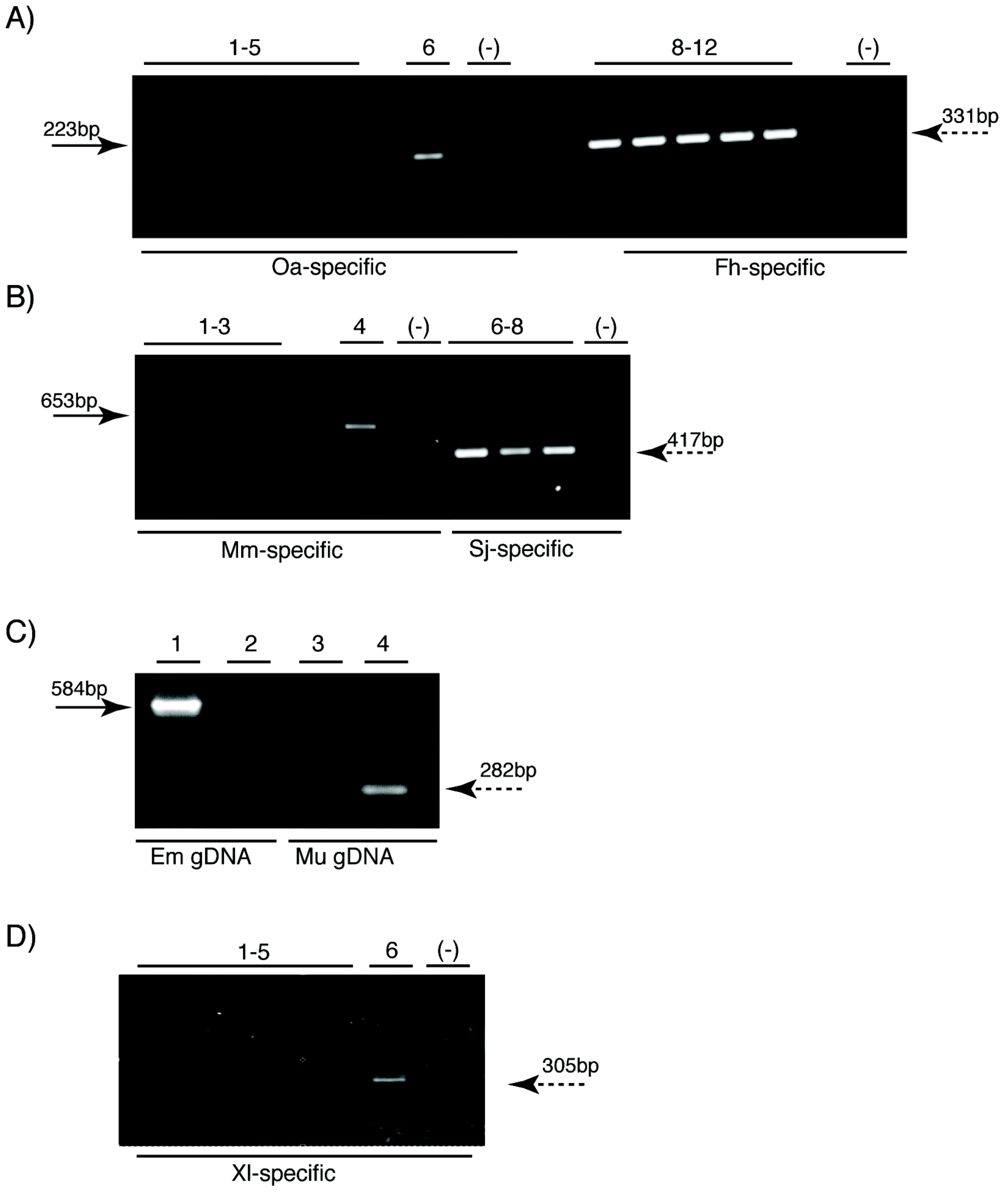

gDNA samples derived from *S. japonicum*, *F. hepatica*, *E. multilocularis* and *P. xenopodis* are free from host contamination. (-) represents non-template controls (water). **A)** Lanes 1-5 correspond to *F. hepatica* gDNA samples which were screened for host contamination using *Ovis aries* (Oa) -specific primers (OaATPase-F: 5' - TTAAAGACTGAGAGCATGATA - 3', OaATPase-R: 5' - GAAAGAGGCAAATAGATTTTC - 3'; [1]) to amplify a region of the mitochondrial ATPase 8 gene (NCBI accession number AF039171.1). Lane 6 represents the 223bp product derived from a positive control *O. aries* gDNA control sample using the same primers. The amplicons (331bp) in lanes 8-12 were produced using *F. hepatica*-specific RAPD primers ([2]; NCBI accession number AY704404.1; FhRAPD-F: 5' - GCGGCCAAATATGAGTCA - 3', FhRAPD-R: 5' -

CTGGAGATTCCGGTTACCAA - 3') with *F. hepatica* gDNA. **B)** Lanes 1-3 represent *S. japonicum* (Sj) gDNA samples subjected to host contamination testing using *M. musculus* (Mm) -specific primers (MmCatalase-F: 5'- ATGGCCTCCGAGATCTTTTC- 3'; MmCatalase-R: 5' - GGGTCAAAAGCCATC TGTTC - 3') to amplify a region of the catalase gene (NCBI accession number X52108.1). Lane 4 represents the 653 bp product derived from a positive control *M. musculus* gDNA sample using the same primers. The amplicons (417 bp) in lanes 6-8 were produced using *S. japonicum* -specific Tyrosinase1 (TYR1; NCBI accession number FJ617452.1) primers ([3]; SjTYR1-F: 5' - AGAATTGATTGGCCAAAACG - 3'; SjTYR1-R: 5' - ACGATGCCATGTAGGGAAAC- 3'). **C)** Lane 1 represents *E. multilocularis* gDNA amplified with primers specific for an *E. multilocularis* insulin receptor tyrosine kinase gene (*emir)* ([4]; NCBI accession number AJ458426; EmIR-N2: 5' - TCTGCTCGCACGCGCGAGGTAG - 3'; EmIR-UP6: 5' - GGTCTAACAACAAAGGATCAG - 3'). The *E. multilocularis* amplicon (584 bp product) in lane 1 is not observed in a similar PCR reaction where host *M. unguiculatus* (Mu) gDNA is used as a template (lane 3). Lane 2 represents *E. multilocularis* gDNA subjected to host contamination testing using *M. unguiculatus* - specific primers to amplify a β-tubulin gene ([5]; NCBI accession number AJ249551; MuTub12-UP: 5' - CAACGTCCAACACCGAGTCA - 3'; MuTub12-ST: 5' - CTGGGCAGTGCGGCAACCA- 3'). Lane 4 represents the 282 bp β-tubulin product derived from a positive control *M. unguiculatus* (Mu) gDNA sample. **D)** Lanes 1-5 represent *P. xenopodis* gDNA samples subjected to host contamination checks using *X. laevis* (Xl) primers (XlMHC-F: 5' - ATTGCAGGGAGCTATTCTAATAAC- 3'; XlMHC-R: ACTGTTAGAGCTGTCACCAGG- 3') specific to a region of CD74 (NCBI accession number NM_001197112.1). Lane 6 represents the 305 bp CD74 *X. laevis* amplicon derived from a positive control PCR reaction containing *X. laevis* gDNA and the same primers.

References

1. Colgan S, O'Brien L, Maher M, Shilton N, Mc Donnell K, Ward S: **Development of a DNA-based assay for species identification in meat and bone meal**. *Food Research International* 2001, **34**(5):409-414.
2. McGarry JW, Ortiz PL, Hodgkinson JE, Goreish I, Williams DJ: **PCR-based differentiation of *Fasciola* species (Trematoda: Fasciolidae), using primers based on RAPD-derived sequences**. *Ann Trop Med Parasitol* 2007, **101**(5): 415-21.
3. Fitzpatrick JM, Johansen MV, Johnston DA, Dunne DW, Hoffmann KF: **Gender-associated gene expression in two related strains of *Schistosoma japonicum***. *Molecular and Biochemical Parasitology* 2004, **136**(2):191-209.
4. Konrad C, Kroner A, Spiliotis M, Zavala-Gongora R, Brehm K: **Identification and molecular characterisation of a gene encoding a member of the insulin receptor family in *Echinococcus multilocularis***. *International Journal for Parasitology* 2003, **33**(3):301-312.

5. Brehm K, Kronthaler K, Jura H, Frosch M: **Cloning and characterization of b-tubulin genes from *Echinococcus multilocularis*.** *Molecular and Biochemical Parasitology* 2000, **107**:297-302.
