# Supplementary material for: Cytosine methylation is a conserved epigenetic feature found throughout the phylum Platyhelminthes
Source: BMC Genomics. 2013 Jul 9;14:462. doi: 10.1186/1471-2164-14-462 (PMC3710501; doi:10.1186/1471-2164-14-462)
Supplement: Additional file 3 — Fasta sequences of retrieved platyhelminth Dnmt and MBD homologs. [file 1471-2164-14-462-S3.pdf]

Additional File 3
Fasta sequences of retrieved platyhelminth Dnmt and MBD homologs

NCBI database

Neobenedenia melleni Dnmt (NmDnmt; GW920589.1)

```
>Nm_Dnmt
MKTMEINVAELFSGIGGMRLALKKSTLQFNVLQAYDIHDGANDIYNYNFKEKCAKNRDIK
ALHCEESVLKRCELWTMSPPCQPYTRLGNMKQSKDTRSEPFAHVIELIRKMSPKLIFLEN
VQGFEESDSHEVLISCLIEMSYDIKQYLLTPLQFGVPNCRTRFYLIAKLSPETPSLKIKT
TLDNNLPRLPGCCCKYCSTEYNESGEEITNYLECNENLNFVKDEDLYRYFRAMDVVSPKD
SKTKCFTKGYSKRFTGTGSFLQCCENPIKHDDIDEKRNSF
```

Paragonimus westermani MBD (PwMBD; AT007407)

```
>Pw_MBD
MLNHQSNQPKRNVYINVPKISTAVAPPCTSNGHSQVIPISLPAGWRREECTRPSGLGTGK
TDVFYMSPSGQKFRTKQEMKAVLGDACDLTLFDWRSGKFLAPSQKVRRPDDGSDPTASKV
PRVDGCSVLTRRTNPPETFVPVIIRSHPECKRTDVRNAQHETPHQLFWEKRFTGKHAVDP
DTGEALKPLNLPNTIQTAGVPGYQPAQMVQSLVNAIAS
```

Schistosoma japonicum Dnmt (SjDnmt; FN238874 & FN328873)

```
>Sj_Dnmt
MRVLELYSGIGGMHFAFQMSTLKHEVVAAIEINDIATDVYKHNFPNTLTLNRVIESFSAN
SLCSFNSNVWSLCPPCQPFTRLGKRMCEVDNRSSSFFHVLDLISILKPEGIILENVKGFE
HSEPWRRLMKVLNACDYEYRQFLLSPLQFGIPNCRLRFYLVARSSSSSWNSNLKMGKSES
IDLRPPIDSPVLPGCRCASCCGLVSHIEHTDDKFTDYIQFCLPVSEFLLTANDREELNFL
DRKCLERYFRVLDIVRSCDKKTRCFTKGYSKRLEGTGSVIQTSMEDETGDKITSYYESNK
ENKQAVLQYAEQLKLRFFHSREVANMLCFPKRFDFPERVTEKQRLRLLGNSVNVLVVSHL
I
```

Schistosoma japonicum MBD (SjMBD; AAW26585.1)

```
>Sj_MBD
MMSVTGDNKLCIMQNQSIPANQLNNSQTKRSSYSNYNKLQTSTSSSPQQQQQTWPSSLPP
GWKREECMRPNGLGTGKSDVYYISPQNQVVRNKQEMQTILGDKYDIASFDWRIGKFNMNN
NNKSKRLEEVTADTSAAAKVPRLDNQYNLPVRRCLFSSTTNEIKPVIVRSHPECKRTDVK
NTNHEIPHQLFWEKRLADHMAINPDTGEPFKPISLPKGMQCRRILAGVPGYQPTQLIQSV
SHALSSSSSNNNSSPIIGQEQQPSAIEKNPCVIINHLQPMIKTFIVTDDDIRRQEARVKE
LRKKLETARKKLHPRYETEREG
```

WTSI GeneDB (http://www.genedb.org/)

Echinococcus multilocularis Dnmt (EmDnmt; EmuJ_001185500)

```
>Em_Dnmt
MRVLELYSGIGGMRAALELSGLAFEIARALDVNDLANSVYNSAYNCSDASNRALESLSEE
ECLLFGADLWTMSPPCQPFTRMGNQKRGKDTRCSSLLIIIQLIRRIKPPAVMLENVKGFE
GSDAWCAILEALIACDYDVRFLLTPLQFGIPNCRLRYYLVARLRTHNKQRMFSFGTSVDD
LKGSFEKLASAIIRTPPCDVSPMPNCECGVCTNKVGGIASTENHFLEYIPFCHPIADYLL
PECEIPEDLFLGQNELEKYYTILDIVTPENRKSACFTKGYAQRFEGTGSFLEVPQPSRSN
STSFPKIRAFHSKEIARLLCFPEYFDFPATVSEKQRRRLLGNSLNVLVVAHIMNWAFSTV
```

Echinococcus multilocularis MBD (EmMBD; EmuJ_001033700)

```
>Em_MBD
MATHPKYVSQSPLSVNKQIAQNMMFQKVTINHTVPITQKSASALPVGWKREEILRPDGLS
SGKTVVFYVSPNGMKIRTQKELQNALGDKFDISSFDWRTGKFSSSLLKHRIPNSGDQNSV
KSSFFRSITSSECKTEDIEYDWFQRNTTPNFPEPVIVLNHNDSNRSDKSPVKESLKQLFS
ERRLSDRYAVDHRDGETLIRSTLPPGIESAGVPGYNKSQLLQNLIVSLSSKHGPITGQER
TVEQNPCAMLNQNQPYVKNFVITDEDIRKQYQRVKDIRDRLQAARKVYPEFLFDLSLSRW
LQTAIKPSVSSFTIDASGTPSTLYIQFVHIVVGNRFVSVRRH
```

Echinococcus granulosus Dnmt (EgDnmt; EgrG_001185500)

```
>EgDnmt
MRVLELYSGIGGMRAALELSGLPFEITRALDVNDLANSVYNSAHNCSDASNRALESLSEE
```

```
ECLLFGADLWTMSPPCQPFTRMGNQKRGKDTRCSSLLIIIQLIRRIKPPAVILENVKGFE
GSDAWRIILEALIACDYDVRFLLTPLQFGIPNCRLRYYLVARLRTHSKQRMFSFGTSVDD
LKGSFEELASAVIRTPPCDVSPMPNCECVVCTNKVGGIASTENHFLEYIPFCRPIADYLL
PECEIPEDLFLGQNELEKYYTILDIVTPENRKSACFTKGYAQRFEGTGSFLEVPQPPHSN
STSFPKIRAFHSKEIARLLCFPEYFDFPATVSEKQRRRLLGNSLNVLVVAHIMNWAFSTV
```

Echinococcus granulosus MBD (EgMBD; EgrG_001033700)

```
>EgMBD
MATHPKYVSQSPLSVNKQIAQNMMFQKVTINHAVPITQKSASALPVGWKREEILRPDGLN
SGKTVVIYVSPNGMKIQTQKELQNALGDKFDISSFDWRTGKFSSSLLKHRIPNSGDQNSV
KSSFFRSITSSECKTEDVEYDWFQRNTTPNFPEPVIVLNHNDSNRSDKSPVKESLKQLFS
ERRLSDRYAVDHRDGKTLIRSTLPPGIESAGVPGYNKSQLLQNLIVSLSSKHGPITGQER
TVEQNPCAMLNQNQPYVKNFVITDEDIRKQYQRVKDIRDRLQAARKVCPEFFFDLSLSRW
LQTAIKPSVSNFTIDASDTPSTLYIQFVHIVVGNRFVCVRRH
```

Hymenolepis microstoma Dnmt (HmDnmt; HmN_000063350)

```
>HmDnmt
MRTLEFYSGIGGMHAALKTSGLPHEVIKAFEINDSANSIYNEVFDCSYATNRTIESLSEM
ECLAYDAELWTMSPPCQPFTRLGNKKQGLDSRSSSLYNIIDLIKKIKPPVIFLENVKGFE
GSDIWKKLIETLILCNYDIRQFLLTPLQFGVPNCRLRYYLVAKLRTNDDQTMFKFGESTT
HLQSFLENIEDNIIRLPIFDVEPMPGCDCVVCTKQVKHIDSIENDYEQYLPFCSPVSSYL
LPDNELPEDIYLGQDILKKYYRILDIIEPESHKTACFTKGYGRRYEGTGSFLSLSKVGDI
GEHHFPQMRRFHSKEVAKIMCFPDNFEFPPNFTEKQRLKLLGNSINVRVVAHIMHWTLSP
```

Hymenolepis microstoma MBD (HmMBD; HmN_000163900)

```
>HmMBD
MAQFKSPSTPVPRPKMEADQQILINRLKTIKLNFITMIQKTGFNHQSSAIQKTLSVLPSG
WRREEVIRPSGLNTGKNLVFYVSPDGKTYKTLKELQLGLGSKYDLSTFDWRTGKFLTTSL
KYKVPGNEDHVNHKRSKPYDMDNDWFQRGSAIKFPKIELKSNHGLSKRIDKPEAQECLRQ
LFAERRLFDRCPVDHRDRKTKIEATLPPGVESCGVPGYNKMQLMQNLIVSLYTKSGPISG
QERCVEQNPCAMINQNQPFVKSFIVIDEDVRKQQHRIAAEKQEF
```

The Taenia solium Genome Project (http://www.taeniasolium.unam.mx/taenia/)

Taenia solium Dnmt (TsDnmt; TsM_000460600 with added/altered exons from genomic scaffold TsM1_s601**)**

```
>TsDnmt
MRILELYSGIGGMRAALELSGLPFKIIKAFDINDLANFVYNSVYKCSDASNRALESLSEA
ECLLYDADLWTMSPPCQPFTRMGNQKRGQDVRCSSLLVVIHLIRRTKPPAILLENVKGFE
GSDAWRYILDALIECDYDVRQFLLTPLQFGIPNGRLRYYLVARLRTRSEQQMFSFGDSVD
LNGCLREPTSAIMRTPPCGVSPMPNCECPVCTNKIRQVLLRYMRFFVSFSDMVSPENHFL
EYLPFCRPIADFLLTDLEIPESLFLGQNVLEKYYTVLDIVTPESRKSACFTKGYAHRFEG
TGSFLEMSQSSHSGLTPFPKIRAFHSKEIARLLCFPEHFDFPEEVSEKQRRRLLGNSINV
LVVAHVMNWTLSTV
```

Taenia solium MBD (TsMBD; TsM_000235700 with altered exon based on cestode MBD gene structure**)**

```
>TsMBD
MPITQKSVSALPVGWKREEILRPDGLNSGKTIVFYVSPSGMKVRTQKELQNVLGDKFDIS
SFDWRTGKFSSPLLKHRVPNLGEQSSVKKCKTEDAECDWFQRNTTPNFPKPVIVLNHTDS
NRSDKSPIKESLKQLFSERRLSDRYAVDHRDGKTLMRSTLPPGIEAAGVPGYNKSQLLQN
LIVSLSSKHGPITGQERTVEQNPCAMLNQNQPYVKNFVITDEDIRKQYQRVKDIRDRLQA
ARKVTSSGHQP
```

SchistoDB beta version (http://beta.schistodb.net/schistobeta/)

Schistosoma haematobium Dnmt (ShDnmt; Sha_108347 - added exon positions from Scaffold471 319482 – 319631, 319663-319768bp)

```
>Sh_Dnmt
MRVLELYAGIGGMHIAFKGSTVKHEVVAAVEINDVATDVYKYNFPNTLTLNRVIESFSPDY
VCSLNANIWSLCPPCQPFTRLGKRMCEADKRSSSFFHVLDLISILKPTGIILENVKGFEH
SEPWRQLIEVLNSCDYEYRQFLLSPLQFGIPNCRLRFYLLARLRSSSWNSNFKMGQSESI
DMRPPVDAPMLPGCQCTSCSGVISHIEHTDDNFTEYIQFCRPISEFVLVPSDSPKELYFL
DEKCLQRYFRVLDIVRSCDKKTRCFTKGYSKRLEGTGSVFQTSMENEICVNYRLNHKQRV
DKFSLEIFQSMLTSEKITNYYEANKEDEQAVLQYAKLLKLRFFHSREVANMMCFPKSFDF
```

PEHITEKQRLRLLGNSVNILVVSHLIYWVFGT

SmedGD v1.3.1.4 (http://smedgd.neuro.utah.edu/)

Schmidtea mediterranea Dnmt (SmdDnmt; mk4.000626.03.01)

```
>Smd_Dnmt
MKVIEFFSGIGGIKCALNGNILSNAISLTFDINDLANSVYRYNFTSPCYNKVIESISAEY
LISLNADMWTMSPPCQPYTRNGNMMDLDDPRTAAMKHTLYLISQVRPHYIFFENVKGFES
SNGRKMLVSILSESAYSFQSQEFLLSPLQFGVPNSRLRYYLIAKLEGKGNLMQDLEAISY
KPYFDRKLYQCNCPVCSGRSRSLENDHVNHFERNLEFCDRISAYLEADNLAEPHEGHKLI
DEKVLEKGFSKLDIVTESSNKTCCFIKCYAKKIEGSGSYYQMTSCEEAHQLKQLLLNGDI
SSLDYAKRLQLRYFSPREIANFMCFPQSFRFPETVTRAQRYRLLGNSVNVKVVAHVLHWL
ISA
```

Schmidtea mediterranea MBD (SmdMBD; mk4.000465.10.01)

```
>Smd_MBD
MSKQSFPSNTNLNSKKANSNATQPTRTSVFNATAVNHQTTSLPPGWKREEILKPTGLYAG
KTDIYYTSPTGQKIRSKAEMQKILGGSYDVSNFDWHSGKFVNSRKRPSTNENDSTSCKLM
KMIEEYQQCLVRSGPSLFCKDKLPIIETTRPDSRINNDAVGGNDNQEVPKQIFWHKRLAD
NYAFNPNTNEKVRSVELPKEFQNAGFPGIDNNQLFQSIVSCIYNSTKNNIPVLGQSNHKA
SIEKNPCLLITANQPLVKQVVITDDIINRQEAKVKELRRKLELVRRTLLPKLSVDEAA
```

Gasser Laboratory database (http://www.gasserlab.org/) & NCBI sequence Read Archive (http://www.ncbi.nlm.nih.gov/sra)

Clonorchis sinensis Dnmt (CsDnmt; Contig6449)

```
>Cs_Dnmt
MRVLELYSGIGGMHCAFKMA
QLDFQVVAAIDINDTANRVYRANFAHTYAPNRVLESLSIEEVTAFNADMWSMSPPCQPFT
RLGNQKHEEDNRSASFFYVLGLIAAIRPKFILLENVKGFEHTEPWCQFLKVLQTCGYRYQ
QFLLTPLQFGVPNCRLRYYLVASSSSDSACSGLFASHVHDSDHANTIHLIPPADLPPLPG
GECAVCLGHVSHITKPDENFDDYLPYCRPISDYLAQQQQHSKLDFLDHNCLKRYFHVLDI
VRPCDRKSRCFTKGYQKRIEGTGSVLQTATDTLTSEEINAQWITARSDPETLMSLAKRLQ
LRFFHSREVANLLCFPQTYNFPEDVTEKQRIRLLGNSVNVLVVAHLIHWAFGDLSHX
```

Clonorchis sinensis MBD (CsMBD; Contig1306)

```
>Cs_MBD
MLNSQPSGTHSSIGQQKRNTYTHLSKGQSQPHSIVPGGHSVPTFIGLPSGWKREECTRPC
GLSTGKTDVYYISPNGQKVRTKHEMKFLLDDAYDISTFDWRTGKFQMSASKVRRTDDTSS
PTSAKVPRMDSAFVRRTSPPDVFTPIVVRSHPECKRTDXQEPQPGASSSAXFGKNVLKTL
MQSIQKQVRLFKQLSLPKDIQSAGVPGYEAPQLVQSLVNALATKSSPITGQEQPVSAFEK
NPCVAANNLQPMIKTYIVSDEDVRRQEARVRELRRKLELARKKLNPRYSSERHXVEQLDA
EWPERVLAPNSHCTYACLSVPVYNAYLVALIVLIVQLPHVYTMQNCNLANLHSGYFLGQM
HELCCFEQLQSDVGYPWL
```

Opisthorchis viverrini Dnmt (OvDnmt; Contig11296)

```
>Ov_Dnmt
MRVLELYSGIGGMHCAFKMSQLDFQV
VAAIDINDTANRVYRANFSHTHAPNRVLESLSIEEVTSFTADMWSMSPPCQPFTRLGNQK
YEEDNRSTSFFYVLGLIAAIRPKFILLENVKGFEHTEPWRQFLKVLHNCGYRYQQFLLTP
LQFGVPNCRLRYYLVASSSSDSASSGLFAPHVHDSSHANTIHIVPPADLPPLPGCECPVC
LGHVNHITKPDENFDEYLPYCRPISDYLTQHQQHSNLDFLDDSCLRRYFHVLDIVRPCDH
KSRCFTKGYQKRIEGTGSVLQTATDPLTGEEIKRPMDNCPSDPETQMSLAKRLQLRFFHS
REVA
```

Opisthorchis viverrini MBD (OvMBD; Contig280)

```
>Ov_MBD
MLNSQPSGTHSNIGQQKRNTYTHLSKGQSQPHSILPGGHSVPTFIGLPSGWKREECTRPC
GLSTGKTDVYYISPNGQKVRTKHEMKFLLDDAYDISTFDWRTGKFQMSASKVRRTDDSSS
PTSAKVPRMDSAFVRRTSPPDVFTPIVVRSHPECKRTDVKNLNQEPPHQLFWEKRFKGTY
AVDPETGEAFKQLSLPKDIQSAGVPGYEAPQLVQSLVNALATKSSPITGQEQPVSAFEKN
PCVAANNLQPMIKTYIVSDEDVRRQEARVRELRRKLELARKKLNPVIRVNDTDRTIGMT
```

***Fasciola hepatica* genome database (http://worm1.liv.ac.uk/.)**

Fasciola hepatica Dnmt (FhDnmt; genome sequence (scaffold15595) used with one Gasser Laboratory database transcript transcript (Fh_F hep_A c30799) used to confirm.

```
>Fh_Dnmt
MRVLELYSGIGGIHCALKKSKLSYSIRIAIDVNDLASSVYRHNFPQSPASNRVIEGLSIEEVSRMSADLWTMSPPC
QPFTRLGNRRDEADNRSVSFFHVLTLIAAIRPSFIFLENVRGFESSESCRELLKTLDSNEYLYQQFLLTPLQFGIP
NCRLRFYLVAWRGPTCTNPFKTDDSSLCILDTPPIHLPPLPDCVCSVCNGCVRNVTANNDNFSEYLPYCQPVANFV
HSLNEHEEKSCSITSDCLKRYFYVMDIVRPCDRKTRCFTKGYTKRIEGTGSVLQTSRLDLTSDLIHSNYQSVEHDS
DALVSFTRTLGLRFFHSREVANLMCFPSDYEFPENVTEKQRIRLLGNSVNVHVVAHLMFWAFGDRNFPIKPT
```

Fasciola hepatica MBD (FhMBD; genome sequence (scaffold35919) used with Gasser Laboratory database transcripts (FhContig4567 and FhContig3923 used to confirm.

```
>Fh_MBD
MQPQNVSGHPNLMQPKRNTYLTFPKTQSFSAAMSSNGQQQVMQITLPSGWKREEALRPTGLGTGKTDVYYISPQGQ
KVRTKQEMKMLLGDKYDIHFFDWRSGKFLSQPPKIPTLQKIVQKYHQQKIPHLDTSPNPLQRRTSPPDTIKPVVIR
SHPNCKRADMRNLSMEPPRQLFWEKRLADQVAIDSETNEPCKPLSLPRGVQSAGVPGYRSPQLINSLLYAIATKTS
PIAGQEQAPSAIEKNPCVAVNTLQPMIKTYIVSEEDIRRQEMRVKELRRKLEMARKRLHPRYATEREG
```

***Macrostomum lignano* genome initiative (http://www.macgenome.org/)**

Macrostomum lignano Dnmt (MlDnmt: RNA815_29248 and RNA815_19240 with the genome sequences (scf25220075753149 & deg 2520075300551) used to confirm

```
>MlDnmt
MHQGGGEFRVAEFFSGIGGMHCALQLLRQLVGEREFSYRIAGAFDVNNIANSVYERNFPGVRVCTTSLDSIGLARI
RALAANLWLMSPPCQPFSRLGNRRGLDDVRCRPLLRLLEVLASLPDNERPSRLLLENVQGFEASPAAERLLECLNK
CGYEYRQLLLSPLQFGVPNSRLRFYLLAWRPGAPEPAAARLLPANCLIDRPPSAATSQPLLPSCCCPVCSGRLQPG
QLTHDSHLRCYRPFARPLSDYLSPGADAEPELYLTSRQLEQFFPVLDIRQPRSRATCCFTKGYGKRVEGTGSVLQT
RRLAFGKPSHDRVKAGACPPRTHGEVSHADSAAAAIDASCSSLAPDDLSDDDINNGEFVDSAGGSGDAETMQAYAE
SLGLRYFSAAEVAGLMHFPPGYSFSTSLCLSRRQAYQLLGNSVNVLVIAHLLSYLLLS
```

Macrostomum lignano MBD (MlMBD; RNA815_4367 and RNA815_18637 with the genome sequences (scf250075747452) used to confirm

```
>MlMBD
WIEKVFFLCLEQKIQRSTKPVSMATNLTSVVGLPPGWKREESLKQSGLSSGKTEVYYVSPGGRKLRNRVELQRALG
DRYDLSNFDWRTGRFHPYARPQQHQHQHHHQHHQQASLQSAGRIKTPADMLSSAAAAAAAAAAAA
```
